# Using the K/BxN mouse model of endogenous, chronic, rheumatoid arthritis for the evaluation of potential immunoglobulin-based therapeutic agents, including IVIg and Fc-μTP-L309C, a recombinant IgG1 Fc hexamer

**DOI:** 10.1186/s12865-019-0328-6

**Published:** 2019-12-04

**Authors:** Bonnie J. B. Lewis, Jade Ville, Megan Blacquiere, Selena Cen, Rolf Spirig, Adrian W. Zuercher, Fabian Käsermann, Donald R. Branch

**Affiliations:** 1 Department of Laboratory Medicine and Pathobiology, University of Toronto, 67 College St., Toronto, Ontario M5G 2M1 Canada; 2 Centre for Innovation, Canadian Blood Services, 67 College St., Toronto, Ontario M5G 2M1 Canada; 3 School for Biology-Biochemistry-Biotechnology, Catholic University of Lyon, 10 place des Archives, 69288 Lyon Cedex 02, France; 4 CSL Behring, Research, CSL Biologics Research Center, Wankdorfstrasse 10, 3010 Bern, Switzerland; 5 Department of Medicine, University of Toronto, 67 College St., Toronto, Ontario M5G 2M1 Canada

**Keywords:** Recombinant Fc hexamer, Fc-μTP-L309C, Intravenous immunoglobulin, IVIg, Subcutaneous immunoglobulin, SCIg, Rheumatoid arthritis, RA, Autoimmune disease

## Abstract

**Background:**

High-dose intravenous immunoglobulin (IVIg), and more recently, subcutaneously-delivered Ig (SCIg), are used to treat a variety of autoimmune diseases; however, there are challenges associated with product production, availability, access and efficacy. These challenges have provided incentives to develop a human recombinant Fc as a more potent alternative to IVIg and SCIg for the treatment of autoimmune diseases. Recently, a recombinant human IgG1 Fc hexamer (Fc-μTP-L309C) was shown to be more efficacious than IVIg in a variety of autoimmune mouse models. We have now examined its efficacy compared to IVIg and SCIg in the K/BxN mouse model of endogenous, chronic rheumatoid arthritis (RA).

**Result:**

Using the serum-transfer K/BxN model and the endogenous autoimmune model, amelioration of the arthritis was achieved. Effective treatment required high and frequent doses of IVIg, SCIg and Fc-μTP-L309C. However, Fc-μTP-L309C was efficacious at 10-fold lower doses that IVIg/SCIg. Also, arthritis could be prevented when Fc-μTP-L309C was given prior to onset of the arthritis in both the endogenous model and in the serum transfer model.

**Conclusions:**

Our results show that Fc-μTP-L309C is a powerful treatment for the prevention and amelioration of severe, chronic arthritis in a true autoimmune mouse model of RA. Thus, the K/BxN endogenous arthritis model should be useful for testing potential therapeutics for RA. Our findings provide rationale for further examination of the treatment efficacy of immunoglobulin-based therapeutics in rheumatoid arthritis.

## Background

Plasma derived IgG is a major replacement therapy for primary immunodeficiency [1–3] and is a first line treatment for diseases such as immune thrombocytopenia (ITP) [4–9], chronic inflammatory demyelinating polyneuropathy (CIDP) [10] and Kawasaki disease [11, 12]. Additionally, it is used to treat a variety of other autoimmune and inflammatory diseases and neurologic disorders in combination with other therapies or when traditional therapies fail [13–21]. Although both F(ab’)_2_- and Fc-dependent mechanisms have been suggested to be involved in the anti-inflammatory effects of this therapy, research in the field has emphasized that the IgG Fc fragment is crucial for its immunomodulatory properties [22]. Some of the proposed Fc-dependent mechanisms include the blockade of activating Fcγ receptors (FcγRs) [23], the requirement for the neonatal Fc receptor (FcRn) [24, 25], the expansion of regulatory T cell populations [26], the upregulation of the inhibitory receptor FcγRIIB [27] and the modulation of dendritic cell activity [28, 29].

Today, plasma derived IgG is pooled from the blood of thousands of donors and manufactured via chromatographic processes to formulate a highly purified, polyclonal IgG product that is suitable for i.v. (intravenous immunoglobulin, IVIg) or s.c. (subcutaneous immunoglobulin, SCIg) applications [30, 31]. Even though the use of SCIg has increased the patient convenience associated with IVIg treatment, its manufacture still requires highly specialized production facilities with a focus on pathogen safety [31, 32]. Moreover, its supply is dependent on the availability and the collection of human plasma and it is subject to some natural variability. These challenges associated with growing product demand, production and availability have provided incentives to develop various Fc constructs as potential alternatives to IVIg/SCIg for diseases where its mechanism has been suggested to be Fc-dependent [22–29].

Various Fc multimers have been produced that show enhanced efficacy compared to IVIg for amelioration of disease in animal models, such as ITP and arthritis [33–41]. In a recently published paper, we reported a recombinant human IgG1 Fc, Fc-μTP-L309C, which was produced by fusing the 18 aa IgM tail-piece to the C-terminus of a variant human IgG1 Fc with a point mutation at position 309 [38]. This point mutation facilitates the stabilized hexamerization of this molecule through the formation of disulphide bonds and distinguishes this recombinant hexamer from others reported [38]. We showed that Fc-μTP-L309C has high binding avidity for Fc receptors and could suppress acute collagen antibody- and chronic collagen-induced arthritis and ameliorate ITP in mouse models when given therapeutically at 10-fold lower doses than IVIg [38].

With the limited amount of studies performed on the therapeutic efficacy of IVIg in rheumatoid arthritis (RA) [20, 42–47], we decided to examine whether IVIg could ameliorate arthritis in an endogenous, chronic mouse model of RA. The K/BxN serum-transfer model is an established mouse model of RA that recapitulates the effector phase of the disease and has been examined for potential treatment efficacy of IVIg by a number of investigators [48–51]. However, K/BxN mice, themselves, that spontaneously generate a true autoimmune-mediated RA, have never been used as a therapeutic tool to investigate the efficacy of RA therapeutic agents, including IVIg.

K/BxN mice are generated using KRN mice expressing a T-cell receptor (TCR) transgene for glucose-6-phosphate isomerase (G6PI) peptide in the context of IAg7 MHC II [52]. To generate K/BxN mice, KRN are bred with NOD/Lt mice expressing the MHC II haplotype IAg7 that is required to interact with the TCR transgene. This mating results in the generation of a true autoantibody to G6PI [52–55]. G6PI is present on the articular cartilage and thus, immune complexes form to drive the activation of various immune cells [52–55]. Since the sera of K/BxN mice contain pathogenic autoantibodies to G6PI, it can be transferred into naïve mice and arthritic manifestations occur a few days to weeks later [48–51]. However, serum recipients develop arthritis in the absence of the adaptive immune system, which poses serious limitations for examining the intricacies of the human disease. Thus, instead of a serum-transfer model, we used the endogenous K/BxN mice to better recapitulate the autoimmune-mediated RA; the resulting immune-mediated mechanism would include the influence of T-cells, antigen presenting cells and B-cells; a true autoimmune condition. Because no one has previously shown that IVIg could ameliorate the endogenous chronic arthritis in this mouse model, we used a dose escalation and frequency of dosing approach to determine if IVIg had any efficacy in this RA model. We also investigated the therapeutic efficacy of SCIg and Fc-μTP-L309C in this same model to determine efficacy, if any, and whether any of these agents could serve as a potential therapeutic alternative for the treatment of RA.

## Results

### IVIg can treat chronic inflammatory arthritis

It is controversial as to whether IVIg can be used in the treatment of RA; although, only low doses, not immunomodulatory doses, have been examined [20, 42–47]. Previously, in mouse models of collagen-induced arthritis, we showed that Fc-μTP-L309C could ameliorate the arthritis using a 10-fold lower dose than IVIg [38]. However, the K/BxN mouse model is a very robust chronic arthritis model and is considered to be a better model of human rheumatoid arthritis [52–58]; thus, we wanted to test efficacy of IVIg, SCIg and Fc-μTP-L309C in this endogenous, chronic mouse model of RA and evaluate whether this particular mouse model could be useful to evaluate future potential treatments for RA.

**Fig. 1:**
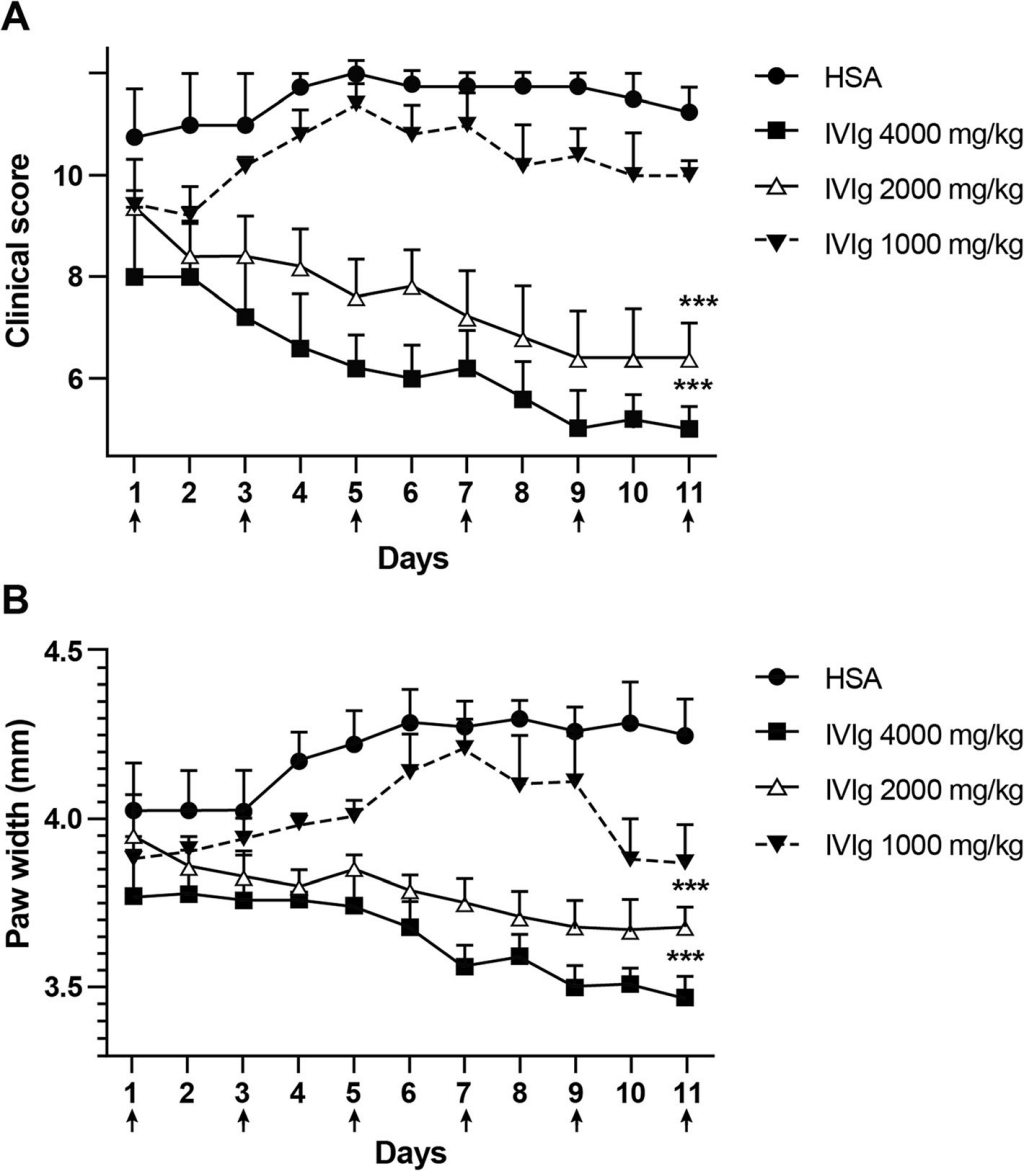
IVIg treats chronic inflammatory arthritis optimally at 2 g/kg doses, administered every other day. The clinical scores (**a**) and paw width measurements (**b**) are shown for mice treated with multiple doses of 4 g/kg, 2 g/kg or 1 g/kg of IVIg, using HSA-treated mice as a control. Arrows indicate treatment given on days 1, 3, 5, 7, 9, and 11. Shown are the average clinical scores and the average paw width measurements (mm); error bars indicate range of clinical scores/range of paw widths (mean ± SD; *n* = 4–5 for each treatment group). **P < 0.001 4 g/kg vs. HSA and 2 g/kg IVIg vs. HSA

We first used a dose-escalation and frequency of dosing approach to determine if there was any effect on endogenous RA and, if so, an optimal dose and dosing schedule at which IVIg exhibited therapeutic efficacy in the treatment of arthritis in K/BxN mice. First, we determined whether there was an optimal dosing of IVIg that would reduce the clinical scores and paw swelling of K/BxN mice with chronic arthritis. We administered 6 treatments with IVIg given i.p. at doses of 1 g/kg, 2 g/kg and 4 g/kg over the course of this experiment. Treatments were administered on days 1, 3, 5, 7, 9, and 11. Clinical scores and hind paw widths of the mice were monitored and it was shown that the clinical scores and paw widths of the mice treated with multiple doses of 4 and 2 g/kg of IVIg (Fig. 1a & b) were significantly reduced in comparison to mice that were given the same g/kg dose of HSA. However, the clinical scores and paw widths of mice that were treated with multiple doses of 1 g/kg of IVIg (Fig. 1a & b) were not reduced in comparison to mice that were given the same g/kg dose of HSA. Further modifying the dosing regimen was not optimal as the mice tended to rebound with their arthritis with reducing or stopping the dosing (data not shown). As the dosing with 2 g/kg or 4 g/kg gave similar amelioration of the arthritis, we decided that an optimal dose of IVIg could be administered using 2 g/kg every other day.

### Fc-μTP-L309C is more efficacious than IVIg at treating chronic arthritis

After demonstrating that IVIg could effectively ameliorate chronic arthritis at 2 g/kg with doses administered every other day, we next wanted to investigate whether SCIg and Fc-μTP-L309C could do the same or better. We administered 6 s.c. treatments of SCIg at 2 g/kg or 6 s.c. treatments of Fc-μTP-L309C at 200 mg/kg, 100 mg/kg or 50 mg/kg over the course of this experiment. We started with a dose of 200 mg/kg for Fc-μTP-L309C because of our previous work showing this to be an optimal dose for amelioration of ITP and collagen-induced arthritis [38].

**Fig. 2:**
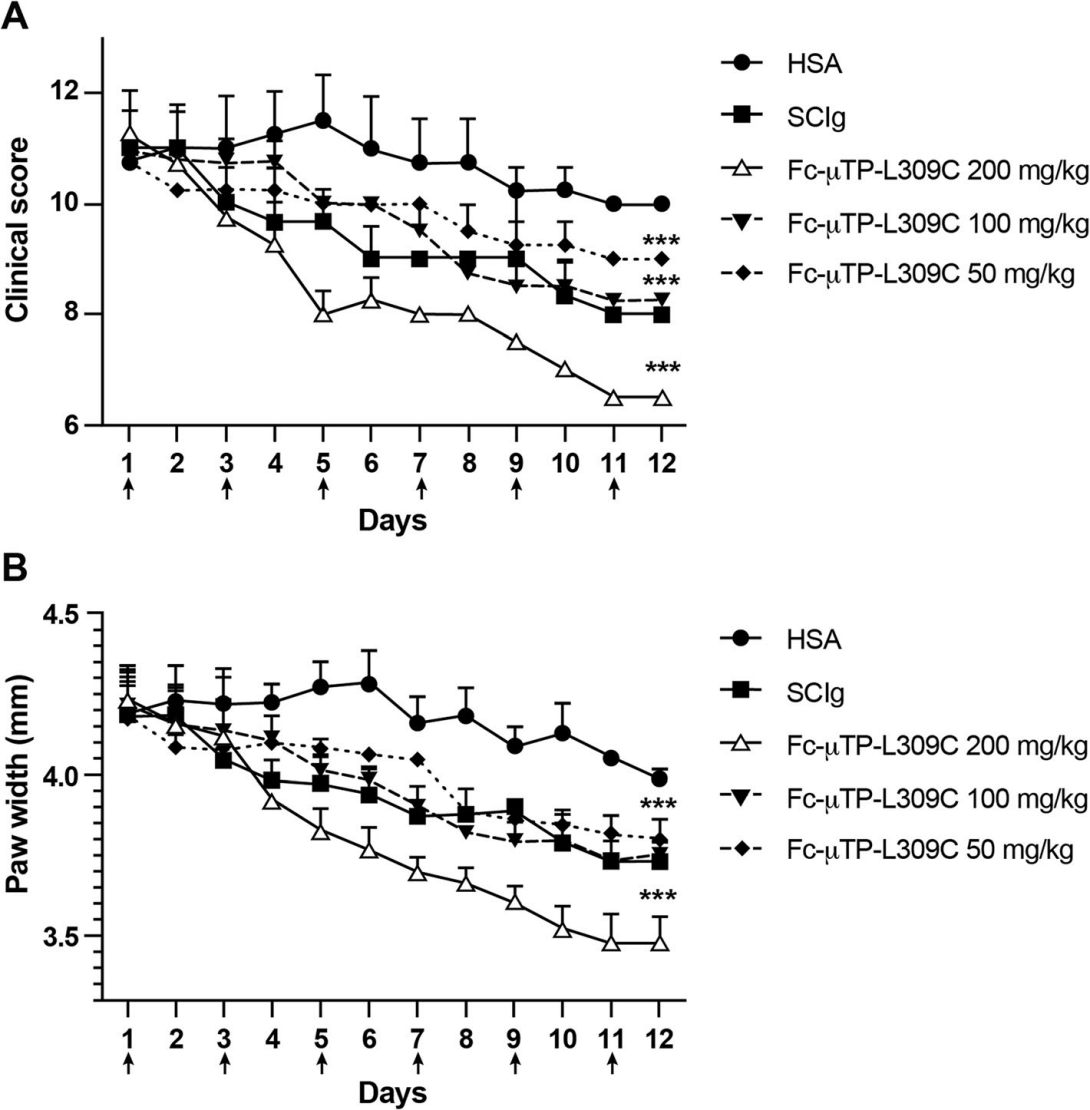
Therapeutic effect of Fc-μTP-L309C in experimental arthritis.The clinical scores (**a**) and paw width measurements (**b**) are shown for mice treated with multiple doses of 2 g/kg of SCIg or with multiple doses of 200 mg/kg, 100 mg/kg, or 50 mg/kg of Fc-μTP-L309C, using HSA-treated mice as a control. Injections were given on days 1, 3, 5, 7, 9, and 11. Shown are the average clinical scores and the average paw width measurements (mm); error bars indicate range of clinical scores/range of paw widths (mean ± SD; *n* = 5 for each treatment group). ***P < 0.0001 200 mg/kg Fc-μTP-L309C vs. 2000 mg/kg SCIg

Treatments were administered on days 1, 3, 5, 7, 9, and 11. We monitored the clinical scores and hind paw widths of the mice and found that the clinical scores and paw widths of the mice treated with multiple doses of 2 g/kg of SCIg (Fig. 2a & b) were reduced in comparison to mice that were given the same g/kg dose of HSA. However, we also found that the clinical scores and the paw widths of mice treated with multiple doses of 200 mg/kg of Fc-μTP-L309C (Fig. 2a & b) were significantly reduced in comparison to mice that were given 2 g/kg SCIg or the same g/kg dose of HSA. Fc-μTP-L309C exhibited a dose-response as even doses of 50 mg/kg and 100 mg/kg showed a trend for efficacy with 200 mg/kg being the most efficacious. Even though SCIg could ameliorate arthritis with the same dosing scheme as IVIg, Fc-μTP-L309C exhibited greater therapeutic efficacy than both IVIg and SCIg in this model at 10-fold lower doses.

### Fc-μTP-L309C, IVIg and SCIg can prevent arthritis in both the endogenous and in the serum transfer K/BxN model

**Fig. 3:**
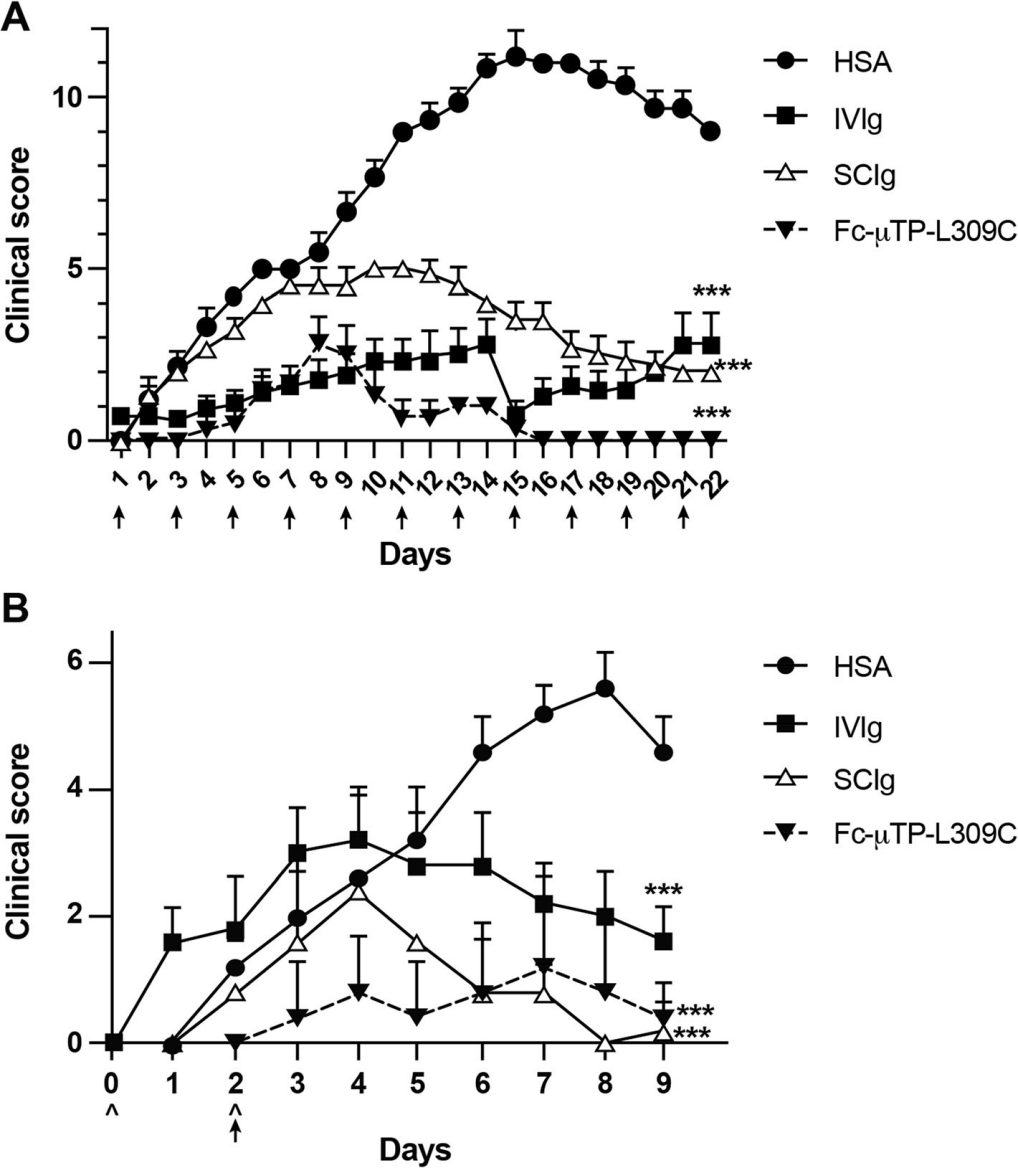
Fc-μTP-L309C, IVIg and SCIg can prevent arthritis in both the endogenous and in the serum transfer model. The clinical scores (**a**) are shown for mice treated with 11 injections of IVIg, SCIg or Fc-μTP-L309C. Injections were given on days 1, 3, 5, 7, 9, 11, 13, 15, 17, 19, and 21. Shown are the average clinical scores; error bars indicate range of clinical scores (mean ± SD; *n* = 6 for each treatment group). ***P < 0.0001 2000 mg/kg IVIg vs. HSA, 2000 mg/kg SCIg vs. HSA and 200 mg/kg Fc-μTP-L309C vs. HSA. Similar results were obtained in two independent experiments. The clinical scores (**b**) are shown for BALB/c mice given i.p. injections of 200 μl of arthritic serum on days 0 and 2, indicated by ^, that were treated with 2 g/kg of IVIg or SCIg or with 200 mg/kg of Fc-μTP-L309C on day 2, indicated by arrow, in comparison to mice treated with HSA. Shown are the average clinical scores; error bars indicate range of clinical scores (mean ± SD; *n* = 5 for each treatment group). ***P < 0.0001 2000 mg/kg IVIg vs. HSA, 2000 mg/kg SCIg vs. HSA and 200 mg/kg Fc-μTP-L309C vs. HSA

As a complimentary study to our investigation of the therapeutic efficacy of IVIg, SCIg and Fc-μTP-L309C in the K/BxN model, we wanted to investigate whether these immunoglobulin-based agents could also prevent arthritis. The first approach we used to examine this was to treat 21-day old K/BxN mice with either 11 i.p. injections of 2 g/kg of IVIg or s.c. injections of SCIg or 11 s.c. injections of 200 mg/kg of Fc-μTP-L309C. The clinical scores of the mice were monitored over the course of this experiment. Injections were administered on days 1, 3, 5, 7, 9, 11, 13, 15, 17, 19, and 21. We found that the clinical scores did not significantly increase in mice treated with 2 g/kg of IVIg (Fig. 3a) or SCIg (Fig. 3a) or with 200 mg/kg of Fc-μTP-L309C (Fig. 3a) in comparison to mice treated with HSA. However, the increase in clinical scores of mice treated with IVIg/SCIg were higher than the increase in clinical scores of mice treated with Fc-μTP-L309C. Prophylaxis using SCIg appeared to not be as efficacious as either IVIg or Fc-μTP-L309C early on; however, severe arthritis was prevented. Importantly, prophylactic treatment using Fc-μTP-L309C appeared to completely prevent onset of arthritis, bringing clinical scores to baseline levels. This again highlights the therapeutic efficacy of Fc-μTP-L309C over IVIg and SCIg.

The second approach we used was to examine the prophylactic effect of these immunoglobulin-based agents on the severity of K/BxN serum transfer-induced arthritis. We used Balb/c mice for these experiments treated with IVIg, SCIg and Fc-μTP-L309C. Similar results were found in this model as in the endogenous model (Fig. 3b). However, in this model IVIg appeared to be less able to provide prophylaxis early on but all agents resulted in the inhibition of progression of the arthritis by day 9–10.

## Discussion

The value of IVIg in the treatment of RA has not been extensively evaluated. The human trials that have been done to evaluate this were small and not well controlled, and the results were equivocal [20, 42–47]. The patient cohorts for these studies were small, with some studies having less than 20 patients enrolled [42, 43]. A high-dose IVIg protocol of 1–2 g/kg of IVIg per month for a minimum of 6 months is critical for effectively treating systemic autoimmune diseases [59]. However, these aforementioned studies used lower than immunomodulatory doses of IVIg, used different doses and dosing schedules without any consistent long-term follow-up [42–47]. In mouse models of RA, only collagen-induced and serum-transfer models have been used to evaluate therapeutic efficacy of immunoglobulin-based agents [38, 60, 61]. Given the lack of understanding we have about the therapeutic efficacy of IVIg in RA, we wanted to determine if the K/BxN mouse model of endogenous, chronic rheumatoid arthritis could be used to evaluate potential therapeutic treatments.

We first investigated whether IVIg could treat autoimmune-mediated arthritis in the K/BxN mouse model [56–58]. Other researchers have used the K/BxN serum transfer model to study arthritis whereby the inflammatory response in the serum recipients happens in the absence of the adaptive immune system [48–51]. Although this model is a useful tool to understand how autoantibodies drive the progression of arthritis by interacting with downstream components of the innate immune system, there are some major differences between the serum transfer model and the human disease. These include differences in antibody specificities and function, and differences in immune cells that drive pathogenesis [53, 55]. Considering this, we decided to treat K/BxN mice with severe arthritis endogenously because we, and others, believe that the immune cells involved in driving the inflammatory response in K/BxN mice more tightly mimic those experienced in the human seropositive disease [52, 57, 58].

We are the first to use immunoglobulin-based agents to try and treat the spontaneous generation of RA in the K/BxN mouse model. Therefore, we first used a multiple dose and frequency of dosing approach to determine if there was any effect on the RA and, if so, the optimal treatment regimen where IVIg was effective. We used an established clinical scoring system to determine when the mice had reached a high level of arthritis, which resulted in a clinical score of 10–12 per mouse. Upon reaching a high clinical score, we then treated these mice with 4 g/kg, 2 g/kg and 1 g/kg of IVIg, 2 times per week. With this dosing scheme, we did not observe any decrease in clinical scores or amelioration of arthritis (data not shown). Next, we treated these mice with the same doses of IVIg, administered every other day. With this dosing scheme, we found that the mice that were given 2 g/kg and 4 g/kg doses showed a similar, significant decrease in their clinical scores and in their paw measurements in comparison to mice given HSA. This is something that has not been shown before in this mouse model of arthritis, however it is comparable to results found in the K/BxN serum-transfer model [40, 50] and in the more commonly known arthritis models such as the collagen-antibody-induced arthritis (CAIA) and the collagen-induced arthritis (CIA) models [38], demonstrating the efficacy of IVIg in mouse models of arthritis [38, 48, 49, 51, 62, 63].

Upon establishing the efficacy of IVIg in the K/BxN mouse model, we wanted to compare its effects with those of Fc-μTP-L309C and SCIg. Both i.v. and i.p. injection of IVIg have been used previously without any evidence of a difference in efficacy. Because i.p. is easier and more volume can be injected, we used i.p.in our studies. We observed that SCIg and IVIg were equally effective at ameliorating endogenous arthritis and that Fc-μTP-L309C was more effective than both SCIg and IVIg at 10-fold lower doses. It should be noted that Fc-μTP-L309C also exhibited higher therapeutic efficacy when given intraperitoneally in both the CIA and in the CAIA mouse models of arthritis [38]. In our case, to better insure we would see an effect, we used high doses of Fc-μTP-L309C, due to the higher frequency of dosing, and was based on the serum half-life of Fc-μTP-L309C to be very short [38]. Additionally, it has been proposed that subcutaneous absorption of biotherapeutics is relatively slow and mostly incomplete [64]. These factors also pose potential issues in developing dosing schemes to test the efficacy of Fc-μTP-L309C to treat human diseases.

Lastly, as a complementary study to our work in the endogenous model, we wanted to know if IVIg, SCIg or Fc-μTP-L309C had any prophylactic activity. We used both the endogenous model and the serum transfer model to investigate this. For the endogenous model we treated mice with either IVIg, SCIg, or Fc-μTP-L309C at weaning age before arthritis had developed. In both models, we found that both IVIg and SCIg prevented disease onset equally in these mice with the exception of a small rebound at the start of the treatment cycle in the serum transfer model. Fc-μTP-L309C however, exhibited complete prevention without any rebound at the start of the treatment cycle.

## Conclusions

In summary, we are the first group to show that K/BxN mice, a model for human RA, having endogenous, chronic, severe arthritis can be effectively treated with IVIg, SCIg and with a recombinant protein, Fc-μTP-L309C. This model is a close representation of human autoimmune RA that is superior to the passive antibody-induced serum-transfer model of arthritis, which models only the effector phase of arthritis and does not involve the adaptive immune system. Our work demonstrates the utility of using the K/BxN mouse for evaluating potential therapeutic agents for treatment of RA. Although the efficacy of both IVIg and recombinant Fcs have yet to be confirmed in human studies, our results show that perhaps using higher doses and more frequent dosing in human RA could have benefit. Perhaps immunoglobulin-based therapies would show efficacy if used to treat certain subgroups of patients, or to treat patients with comorbidities that preclude them from using first line therapies, or to treat patients in whom other therapies are contraindicated. Importantly, the therapeutic effects of both IVIg and Fc-μTP-L309C could be achieved with s.c. routes of administration. The s.c. administration of IgG products has become increasingly attractive in recent years due to patient convenience (home administration) and better systemic tolerability. Considering that lower doses are needed for Fc-μTP-L309C to exhibit therapeutic efficacy in comparison to IVIg with the s.c. route of administration, Fc-μTP-L309C, given subcutaneously, could serve as a possible replacement therapy for IVIg/ SCIg in certain autoimmune diseases.

## Methods

### Mice

KRN T-cell receptor (TCR) transgenic mice on a C57BL/6J background were obtained from The Jackson Laboratory (Bar Harbor, ME), a kind gift from C. Benoist (Harvard Medical School, Boston, MA). NOD/LtJ mice were purchased from The Jackson Laboratory. Arthritic mice were obtained by crossing KRN mice (F, 6 weeks old) with NOD/Lt (M, 6 weeks old) mice to produce K/BxN mice expressing both the TCR transgene KRN and the MHC class II molecule I-Ag7. BALB/cJ (F 6, weeks old) were purchased from The Jackson Laboratory (Bar Harbor, ME). Mice were kept under a natural light-dark cycle, maintained at 22 ± 4 °C, and fed with standard diet and water ad libitum. The use of animals was consistent with the requirements of the CCAC – Canadian Council on Animal Care and the specific animal use protocol, AUP 1788.19, was reviewed and approved by the University Health Network (UHN) Animal Research Committee in Toronto. In general, 3 to 6 mice were used in each experimental and control group of animals. The numbers of animals used in each experiment can be found in the corresponding figure legends. To justify the minimum number of animals used for each experiment, we calculated the group size (n) required at 80% power and significance level (two-tailed, alpha =0.05) to observe an effect of the expected size using the *t* statistic and non-centrality parameter (for comparing two means, effect size E/S = k * δ, where *t* value at df = n_total_-2, where n_total_ = n_1_ + n_2_, δ is the non-centrality parameter, and k = (1/n_1_ + 1/n_2_)^1/2^). When the experiments were finished, mice were euthanized by cervical dislocation after CO_2_ inhalation.

### Biological reagents

Privigen 10% IVIg, Hizentra 20% immunoglobulin (IgG) for subcutaneous use (SCIg), and Fc-μTP-L309C, respectively, were from CSL Behring, Research, CSL Biologics Research Center (Bern, Switzerland). Human serum albumin (HSA) was from the Canadian Blood Services.

### K/BxN serum transfer arthritis

Severely arthritic adult K/BxN mice were bled and the sera was pooled. BALB/cJ mice were injected intraperitoneally (i.p.) with 200 μl of pooled sera on days 0 and 2 as indicated in the figure legends. The volume of sera was chosen based on in vivo titration of pooled sera. Mice were given treatment on day 2 with either an i.p. injection of 2 g/kg of IVIg, a subcutaneous (s.c.) injection of 2 g/kg of SCIg or a s.c. injection of 200 mg/kg of Fc-μTP-L309C as indicated in the figure legends. HSA was used as a protein control.

### Arthritis prevention in the K/BxN mouse model of endogenous, chronic arthritis

K/BxN mice, prior to onset of arthritis, at 21 days of age, were treated by i.p. injections of 2 g/kg of IVIg or s.c. injections of 2 g/kg of SCIg or s.c. injections of 200 mg/kg of Fc-μTP-L309C. Treatments were given on days 1, 3, 5, 7, 11, 13, 15, 17, 19, and 21, as indicated in the figure legends. HSA was used as a protein control.

### Arthritis treatment in the K/BxN mouse model of endogenous, chronic arthritis

To investigate the ability of IVIg to ameliorate chronic arthritis, K/BxN mice with high clinical scores of 9 or greater were treated by i.p. injections of either 1 g/kg, 2 g/kg, or 4 g/kg of IVIg as indicated in the figure legends on days 1, 3, 5, 7, 9, and 11. The lowest dose of IVIg that showed efficacy was 2 g/kg and this dose was selected to compare to SCIg while a titration of Fc-μTP-L309C was performed using s.c. injections of Fc-μTP-L309C at 200 mg/kg, 100 mg/kg, or 50 mg/kg. Final comparisons were done using 2 g/kg IVIg and SCIg and 200 mg/kg Fc-μTP-L309C. Treatments were administered on days 1, 3, 5, 7, 9, and 11. HSA was used as a protein control.

### Arthritis scoring

The clinical scores and the hind paw widths of the mice were monitored daily over the course of each experiment. The development of arthritis was assessed daily, and the severity of arthritis was scored for each paw on a 3-point scale, in which 0 = normal appearance, 1 = localized edema/ erythema over one surface of the paw, 2 = edema/ erythema involving more than one surface of the paw, 3 = marked edema/erythema involving the whole paw. The scores of all four paws were added for a composite score, with a maximum score of 12 per mouse. Ankle thickness of the hind paws was measured in millimeters (mm) at the widest point (the malleoli) with the legs fully extended with digital calipers (Manostat, Herisau, Switzerland).

### Statistical analysis

Statistical tests were performed using GraphPad Prism 8 for Windows software. Analyses of differences between sample groups were performed using the tests indicated in the figure legends. Data shown are mean ± standard deviation (SD), unless otherwise stated. *P* < 0.05 was considered statistically significant.

## Data Availability

All data generated or analyzed during this study are included in this published article.

## Abbreviations

CIDP: Chronic inflammatory demyelinating neuropathy
FBS: Fetal bovine serum
FcRn: Neonatal Fc receptor
FcγR: Fc gamma receptor
Fc-μTP-L309C: Recombinant IgG1 Fc hexamer
GVHD: Graft versus host disease
HSA: Human serum albumin
i.p.: Intraperitoneal
IgG: Immunoglobulin-gamma
ITP: Immune thrombocytopenia
IVIg: Intravenous immunoglobulin
MG: Myasthenia gravis
mm: Millimetre
RA: Rheumatoid arthritis
s.c.: Subcutaneous
SCIg: Subcutaneous immunoglobulin
SD: Standard deviation
TCR: T-cell receptor
